# All-optical nuclear quantum sensing using nitrogen-vacancy centers in diamond

**DOI:** 10.1038/s41534-023-00724-6

**Published:** 2023-06-10

**Authors:** B. Bürgler, T. F. Sjolander, O. Brinza, A. Tallaire, J. Achard, P. Maletinsky

**Affiliations:** 1 Department of Physics, University of Basel, Klingelbergstrasse 82, Basel, CH-4056 Switzerland; 2 Laboratoire des Sciences des Procédés et des Matériaux, LSPM, CNRS-UPR 3407, Université Sorbonne Paris Nord, 99 Avenue JB Clément, Villetaneuse, 93430 France; 3 Institut de Recherche de Chimie Paris, CNRS, Chimie ParisTech, Université PSL, 11 rue Pierre et Marie Curie, 75005 Paris, France

**Keywords:** Quantum metrology, Quantum mechanics

## Abstract

Solid state spins have demonstrated significant potential in quantum sensing with applications including fundamental science, medical diagnostics and navigation. The quantum sensing schemes showing best performance under ambient conditions all utilize microwave or radio-frequency driving, which poses a significant limitation for miniaturization, energy efficiency, and non-invasiveness of quantum sensors. We overcome this limitation by demonstrating a purely optical approach to coherent quantum sensing. Our scheme involves the ^15^N nuclear spin of the Nitrogen-Vacancy (NV) center in diamond as a sensing resource, and exploits NV spin dynamics in oblique magnetic fields near the NV’s excited state level anti-crossing to optically pump the nuclear spin into a quantum superposition state. We demonstrate all-optical free-induction decay measurements—the key protocol for low-frequency quantum sensing—both on single spins and spin ensembles. Our results pave the way for highly compact quantum sensors to be employed for magnetometry or gyroscopy applications in challenging environments.

## Introduction

Spin-based quantum sensors can be employed to measure a wide range of relevant physical quantities, including magnetic^1^ or electric fields^2^, temperature^3^, or rotary motion^4^. This abundance of potential observables, combined with their high sensitivity at the nano-scale makes quantum sensors highly interesting for many fields of application, such as life sciences^5^, geological sciences^6,7^, navigation,^8^ and material sciences^9^.

**Fig. 1:**
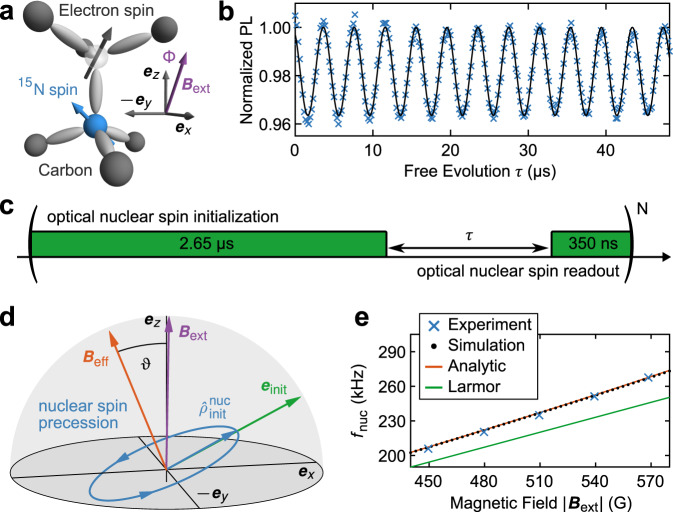
All-optical nuclear magnetometry. **a** Crystal structure of the Nitrogen-Vacancy center with illustration of its associated spins and coordinate axes. **b** All-optical nuclear spin precession of the ^15^N Nuclear spin observed at a magnetic field of ∣***B***_ext_∣ = 540 G tilted away from the NV symmetry axis by Φ = 1^∘^. Fitting of a harmonic function (black) yields a precession frequency 251.18 ± 0.12 kHz. **c** Pulse sequence employed for **b**, consisting of a 3 μs green laser pulse separated by a variable delay *τ*. The first 350 ns of each green pulse are utilized for optical nuclear spin readout, while the remainder of the pulse reinitializes the spin system. **d** Quantitative Bloch-Sphere representation of the ^15^N spin in the $\left\vert {m}_{s}=0\right\rangle$ ground state manifold. For a magnetic field ***B***_ext_ tilted from the NV symmetry axis by the angle Φ = 1^∘^, optical pumping initializes the nuclear spin into ${\hat{\rho }}_{{{{\rm{init}}}}}^{{{{\rm{nuc}}}}}$. The nuclear spin subsequently precesses around an effective magnetic field ***B***_eff_. The measurement axis for all-optical readout of this precession is given by ***e***_init_. **e** Experimentally observed precession frequency (blue crosses) at different values of ∣***B***_ext_∣ and fixed Φ = 1^∘^, together with analytic (solid orange line) and numerical predictions (black dots).

Nitrogen-Vacancy (NV) centers in diamond (Fig. 1a) are a particularly promising platform for such spin-based quantum sensing applications, because they host a single electron spin^10^ with long coherence times^11,12^ even at room temperature^13^. Upon optical excitation with green light, the NV center emits spin-dependent red photoluminescence (PL)^14^, which enables all-optical electron spin readout. At the same time, such optical excitation pumps the NV electron spin^15,16^ into a specific spin eigenstate, enabling all-optical spin initialization. Time-varying (AC) driving fields, mostly in the microwave (MW) or radio-frequency (RF) domain can then be used to coherently control the spin, and create superposition states for sensing. This combination of optical initialization, readout, and coherent spin manipulation by AC driving fields form the basis of almost all established spin-based approaches to sensing^17^.

The NV electron spin is inherently coupled to the nuclear spin of its Nitrogen atom—a spin which exhibits significantly longer coherence times compared to the NV electron spin^18,19^ and therefore provides another interesting resource for quantum technology applications. Specifically, nuclear spins have been exploited as a quantum register for quantum communication^20^ and enhanced spin readout techniques^21^, but they also offer interesting opportunities for sensing, be it for magnetometry^18,22–24^ or for gyroscopy^25,26^.

Many Nitrogen spin-based quantum sensing schemes in diamond rely on the resonant coupling of the NV spin and the nuclear Nitrogen spin at a magnetic field of about 500G^27,28^, where spin flip-flop processes occur in the NV’s orbital excited state at the excited state level anti-crossing (ESLAC). It has been shown that optical pumping in the vicinity of the ESLAC results in nuclear spin hyperpolarization^29,30^, and that—by virtue of the same mechanism—the NV center shows a nuclear spin-state-dependent rate of transient PL^28^. As a result, optical pumping close to the ESLAC enables both all-optical readout of the nuclear spin-state and initialization into a nuclear spin eigenstate, which, together with RF driving, forms the basis for nuclear spin-based sensing schemes^28^.

However, the ubiquitous need for AC coherent driving in spin-based quantum sensing is a severe limitation for many applications. Specifically, such AC driving fields can adversely affect investigated samples, and for integrated or portable sensing devices, their delivery increases power consumption and overall complexity, and thereby size and cost of the system. Recent experiments have demonstrated microwave-free NV magnetometry schemes that are based on sharp changes in NV PL at level anti-crossings, that occur at specific magnetic fields^31,32^. While avoiding the need for MW or RF delivery, these approaches do not exploit quantum coherence (and are therefore limited in sensitivity) and are furthermore highly vulnerable to background drifts in NV PL.

Here, we present a method for coherent, microwave-free quantum sensing using the ^15^N nuclear spin of the NV center in diamond. Our approach is based on optical driving of the NV center near the ESLAC in the presence of a small, static magnetic field transverse to the NV symmetry axis denoted by the unit vector ***e***_*z*_ (see Fig. 1a). As we will show, such a small transverse magnetic field component has a striking effect, in that optical pumping prepares the ^15^N nuclear spin in a coherent superposition state within the NV’s ground state spin manifold. Following optical pumping, this initialization leads to Larmor precession of the nuclear spin about an effective magnetic field; a precession we directly monitor via nuclear spin-state dependent PL^28^. Figure 1b shows an example of such an all-optical nuclear free induction (FID) measurement, obtained using the pulse sequence depicted in Fig. 1c. Data for this work were recorded on a home-built confocal optical microscope with magnetic field control (see methods); here at a magnetic field of strength ∣***B***_ext_∣ = 540 G, tilted by Φ = 1^∘^ away from ***e***_*z*_.

The negatively charged NV center possesses an electron spin *S* = 1 quantized along the NV symmetry axis ***e***_*z*_. For NV centers formed by ^15^N (denoted as “^15^NV” in the following), the Nitrogen nucleus exhibits a spin *I* = 1/2. The Hamiltonian for the orbital ground (gs) and excited state (es) of such a ^15^NV can be expressed as

1 $$\frac{{\hat{H}}^{{{{\rm{gs,es}}}}}}{h}={D}_{0}^{{{{\rm{gs,es}}}}}{\hat{S}}_{z}^{2}+\hat{{{{\boldsymbol{S}}}}}\cdot {{{{\boldsymbol{A}}}}}^{{{{\rm{gs,es}}}}}\cdot \hat{{{{\boldsymbol{I}}}}}+{\gamma }_{S}{{{{\boldsymbol{B}}}}}_{{{{\rm{ext}}}}}\cdot \hat{{{{\boldsymbol{S}}}}}+{\gamma }_{I}{{{{\boldsymbol{B}}}}}_{{{{\rm{ext}}}}}\cdot \hat{{{{\boldsymbol{I}}}}}\,,$$

where $\hat{{{{\boldsymbol{S}}}}}$ and $\hat{{{{\boldsymbol{I}}}}}$ are the NV electron and nuclear spin operators, *γ*_*S*_ = 2.8 MHz G^−1^ and *γ*_*I*_ = 431.7 Hz G^−1^ are the respective gyromagnetic ratios, ${D}_{0}^{{{{\rm{gs}}}}}=2.87\,$GHz and ${D}_{0}^{{{{\rm{es}}}}}=1.42\,$GHz are the zero-field splittings, and ***B***_ext_ is the applied magnetic field. The hyperfine coupling tensor ***A***^gs,es^ has two independent components ${A}_{\parallel }^{{{{\rm{gs}}}}}=3.03\,$MHz and ${A}_{\perp }^{{{{\rm{gs}}}}}=3.65\,$MHz for the ground state; and ${A}_{\parallel }^{{{{\rm{es}}}}}=-57.8\,$MHz, ${A}_{\perp }^{{{{\rm{es}}}}}=-39.2\,$MHz for the excited state^33,34^. This Hamiltonian is conveniently expressed in a basis of spin eigenstates $\{\left\vert {m}_{S},{m}_{I}\right\rangle \}$, where *m*_*S*_ and *m*_*I*_ are the magnetic quantum numbers associated with ${\hat{S}}_{z}$ and ${\hat{I}}_{z}$. The coherent FID dynamics that are studied in this work (Fig. 1b) can be completely encompassed by a reduced subspace spanned by $\{\left\vert 0,-1/2\right\rangle ,\left\vert 0,+1/2\right\rangle \}$.

## Results

### Derivation of an effective Hamiltonian

In the following we explain the nuclear precession data presented in Fig. 1b by discussing how the presence of the transverse magnetic field component *B*_⊥_ significantly affects the system’s optical pumping and subsequent FID dynamics. We start by calculating an effective Hamiltonian for the ^15^N spin in the *m*_*S*_ = 0 ground state subspace, $\{\left\vert 0,-1/2\right\rangle ,\left\vert 0,+1/2\right\rangle \}$, using Van Vleck perturbation theory^35^ (see supplementary methods A for the full derivation). Without loss of generality, we set the transverse magnetic field to point along the unit vector ***e***_*x*_. We then obtain the effective Hamiltonian

2 $$\frac{\hat{H}_{{{{\rm{eff}}}}}^{{m}_{S} = 0}}{h}=\frac{1}{2}\left[\begin{array}{ll}{\gamma }_{I}{B}_{z}+{\nu }_{z}&{\gamma }_{I}{B}_{\perp }+{\nu }_{\perp }\\ {\gamma }_{I}{B}_{\perp }+{\nu }_{\perp }&-{\gamma }_{I}{B}_{z}-{\nu }_{z}\end{array}\right],$$

where

3 $${\nu }_{z}=\frac{{\gamma }_{S}{B}_{z}{({A}_{\perp }^{{{{\rm{gs}}}}})}^{2}}{{({D}_{0}^{{{{\rm{gs}}}}})}^{2}-{({\gamma }_{S}{B}_{z})}^{2}}$$

denotes the correction to the diagonal elements caused by mixing between states of different *m*_*S*_, and

4 $${\nu }_{\perp }=\frac{-2\,{\gamma }_{S}{B}_{\perp }{A}_{\perp }^{{{{\rm{gs}}}}}{D}_{0}^{{{{\rm{gs}}}}}}{{({D}_{0}^{{{{\rm{gs}}}}})}^{2}-{({\gamma }_{S}{B}_{z})}^{2}}$$

is the corresponding correction to the off-diagonal elements. We note that such an effective hyperfine Hamiltonian for ^15^NVs has been discussed earlier as a perturbation in *B*_⊥_ in the limit ${B}_{z}\ll {D}_{0}^{{{{\rm{gs}}}}}$^36^, or as a perturbation in ${A}_{\perp }^{{{{\rm{gs}}}}}$^28^, but never as a perturbation in both simultaneously as we present it here. Additionally, exact analytic expressions for *ν*_⊥_ have previously been derived in Chen et al.^37^.

Hamiltonian ${H}_{{{{\rm{eff}}}}}^{{m}_{S} = 0}$ yields that the ^15^N nuclear spin in the NV ground state is quantized along an effective magnetic field ***B***_eff_. Diagonalization of ${H}_{{{{\rm{eff}}}}}^{{m}_{S} = 0}$ yields

5 $${\gamma }_{I}| {{{{\boldsymbol{B}}}}}_{{{{\rm{eff}}}}}| =\sqrt{{\left({\gamma }_{I}{B}_{z}+{\nu }_{z}\right)}^{2}+{\left({\gamma }_{I}{B}_{\perp }+{\nu }_{\perp }\right)}^{2}}=:{f}_{{{{\rm{nuc}}}}}\,,$$

where *f*_nuc_ is the expected nuclear spin precession frequency. Importantly, ***B***_eff_ is neither aligned with ***B***_ext_ nor with the NV symmetry axis. Instead, it lies in the plane spanned by ***B***_ext_ and ***e***_*z*_, and is tilted away from ***e***_*z*_ by the angle $\vartheta ={\tan }^{-1}\left[({\gamma }_{I}{B}_{\perp }+{\nu }_{\perp })/({\gamma }_{I}{B}_{z}+{\nu }_{z})\right]$. Interestingly, *ϑ* is significantly larger than the misalignment angle Φ between ***B***_ext_ and ***e***_*z*_, and *ϑ* has a sign opposite to Φ due to the negative sign of *γ*_*I*_ (c.f. Fig. 1d). Finally, note that according Eq. (4), *ν*_⊥_ = 0 when *B*_⊥_ = 0, which in turns causes *ϑ* = 0. In this case, both ***B***_eff_ and ***B***_ext_ are aligned with the NV symmetry axis.

### Analysis of ^15^N spin dynamics

We now discuss how the presence of *B*_⊥_ affects the ^15^N nuclear spin dynamics and enables all-optical initialization into a nuclear spin superposition state. The use of ^15^N is key to this, since it does not have a quadrupolar spin splitting which would prevent any nuclear Larmor precession. It is clear from Eq. (4) that when *B*_⊥_ ≠ 0, the ^15^N nuclear quantization axis depends sensitively on the hyperfine coupling parameter *A*_⊥_ and the splitting of the involved spin-levels. Therefore, the ground and excited state nuclear spin quantization axes are in general different, because the hyperfine parameters differ in both magnitude and sign between the two cases. This difference in ^15^N quantization axes results in optical pumping of the nuclear spin into a state that does not correspond to an eigenstate of the effective ground state Hamiltonian ${H}_{{{{\rm{eff}}}}}^{{m}_{S} = 0}$. To be specific, optical pumping accumulates NV excited state population in the eigenstate with the largest *m*_*S*_ = 0 character (i.e. the state $\left\vert \psi \right\rangle$ for which $\left\langle \psi \right\vert {\hat{{{{\boldsymbol{S}}}}}}_{z}/\hslash \left\vert \psi \right\rangle$ is closest to zero), since this state has the lowest probability of shelving into the NV’s singlet state. We denote this state as $\left\vert {\tilde{0}}_{{{{\rm{es}}}}}\right\rangle$. By the same argument, $\left\vert {\tilde{0}}_{{{{\rm{es}}}}}\right\rangle$ is also the “brightest” state in that it yields the largest rate of emission of NV fluorescence photons. For state $\left\vert {\tilde{0}}_{{{{\rm{es}}}}}\right\rangle$, the expectation value of the nuclear spin lies along the vector ${{{{\boldsymbol{e}}}}}_{{{{\rm{init}}}}}=\left\langle {\tilde{0}}_{{{{\rm{es}}}}}\right\vert \hat{{{{\boldsymbol{I}}}}}/\hslash \left\vert {\tilde{0}}_{{{{\rm{es}}}}}\right\rangle$. Vector ***e***_init_ therefore defines both the direction along which the nuclear spin is initialized under green illumination, as well as the measurement axis for optical readout of the nuclear spin. For *B*_⊥_ ≠ 0, ***e***_init_ is not collinear with ***B***_eff_, and thus optical pumping will initialize the ^15^N nuclear spin in a state that is not an eigenstate of ${H}_{{{{\rm{eff}}}}}^{{m}_{S} = 0}$. Disengaging green laser excitation after optical pumping will therefore result in precession of the ^15^N nuclear spin around ***B***_eff_. Finally, note that for NV centers formed with ^14^N, no Larmor precession occurs because its quadrupolar splitting locks ***B***_eff_ onto the NV axis.

### Comparison with numerical results

To further verify this picture, we developed a numerical model that simulates the dynamics of the ^15^NV system during and after optical pumping. The model is based on classical rate equations for the optical pumping process^16^, coupled with master equations describing the quantum-mechanical evolution of the system’s density matrix within each relevant orbital manifold: the orbital ground and excited states as well as the singlet state (see further details in the supplementary methods B).

In Fig. 1d we summarize the numerical and theoretical results in a Bloch-sphere representation of the ^15^N nuclear spin dynamics for the same magnetic field that was used to obtain the experimental results in Fig. 1b. The effective field ***B***_eff_ is calculated numerically through exact diagonalization of the ground state Hamiltonian ${\hat{H}}^{{{{\rm{gs}}}}}$, and ***e***_init_ is calculated via diagonalization of the excited state Hamiltonian ${\hat{H}}^{{{{\rm{es}}}}}$. The nuclear spin density matrix ${\hat{\rho }}_{{{{\rm{init}}}}}^{{{{\rm{nuc}}}}}$ following optical pumping is obtained by propagating the system density matrix $\hat{\rho }$ for 3 μs of laser excitation, followed by a 50ns dark time (to let the system relax fully to the ground state), and by subsequently taking the trace over the NV electron spin degrees of freedom. The nuclear spin precession dynamics are then described by propagating $\hat{\rho }$ under the influence of ${\hat{H}}^{{{{\rm{gs}}}}}$.

We make two observations that underline the excellent agreement of this numerical model with our analytical discussion. First, the initial nuclear spin direction Tr$(\hat{{{{\boldsymbol{I}}}}}\cdot{\hat{\rho }}_{{{{\rm{init}}}}}^{{{{\rm{nuc}}}}})$ obtained from our numerical model is perfectly collinear with ***e***_init_, as long as we set the intersystem crossing rate for the *m*_*S*_ = 0 states to zero (see Supplementary Fig. 3). Second, the orientation of ***B***_eff_ obtained from numeric diagonalization of the full ground state Hamiltonian ${\hat{H}}^{{{{\rm{gs}}}}}$ shows perfect agreement with the analytical prediction from Eq. (2), as well as with the result obtained from the numerical model (see supplementary notes B). Both observations strongly support the validity of our model and its applicability to quantitatively describe our data.

### All-optical nuclear ^15^N precession

The presented theory framework allows us to further analyze the data presented in Fig. 1b. The observed FID oscillation frequency was determined by least-squares fitting to *f*_nuc_ = 251.18 ± 0.12 kHz, in good agreement with Eq. (5), which yields *f*_nuc_ = 252.71 kHz for the experimental conditions ∣***B***_ext_∣ = 540 G and *B*_⊥_ = 10.6 G. Here and elsewhere, the given errors are the standard errors of the coefficients in the respective non-linear regression fits. The small remaining discrepancy can be assigned to uncertainties in controlling the tilt angle Φ, and determining the exact field components *B*_⊥_ and *B*_∥_. To demonstrate that the observed oscillations indeed originate from nuclear spin precession, we repeated the same experiment at fixed angle Φ, while varying ∣***B***_ext_∣. Figure 1e shows the resulting, near-linear dependance of *f*_nuc_ on ∣***B***_ext_∣ and the excellent agreement with the predictions of both Eq. (5) and the numeric model. The enhancement of *f*_nuc_ over the bare Larmor frequency results from the terms *ν*_*z*_ and *ν*_⊥_ in Eq. (5).

### Angle Φ and field ∣***B***_ext_∣ dependance of the all-optical ^15^N FID signal

**Fig. 2:**
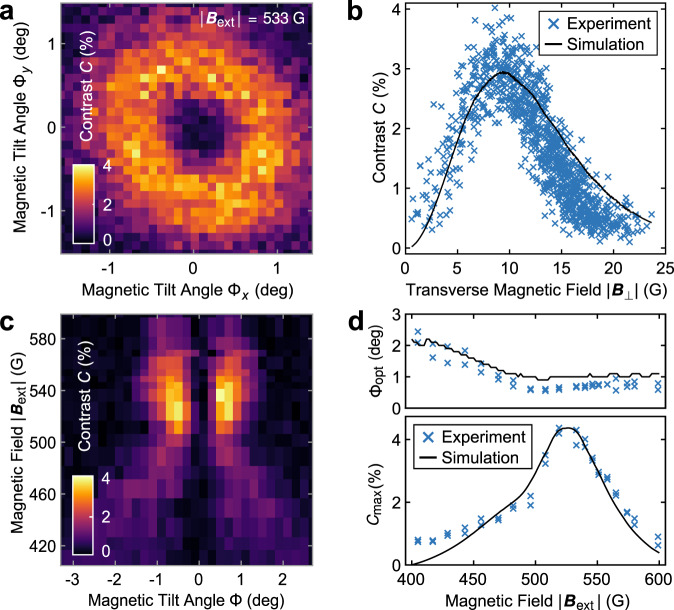
Investigation of nuclear FID contrast. **a** Measured free induction decay (FID) contrast of a single NV as a function of magnetic field orientation, for a fixed total field of ∣***B***_ext_∣ = 533 G. **b** Same data as in **a**, but plotted against total transverse magnetic field *B*_⊥_. The black line is the prediction of our numerical model, which is normalized to the mean of the data at Φ_opt_ ± 0.03^∘^. **c** Nuclear FID contrast as a function of magnetic tilt angle Φ and total magnetic field ∣***B***_ext_∣. **d** Maximal observed contrast ${C}_{\max }$ and corresponding tilt angle Φ_opt_ for each value of ∣***B***_ext_∣. The prediction of our numerical model is shown in black, which is normalized to the maximal data point.

The requirement of applying a transverse magnetic field *B*_⊥_ to obtain an observable, all-optical ^15^N FID signal motivates the question of how the FID readout contrast *C* depends on both Φ and ∣***B***_ext_∣. Figure 2a shows single NV data, where we measured *C* as a function of Φ for a fixed field ∣***B***_ext_∣ = 533G. We determined *C* from the Fourier space amplitude of individual FID curves, for varying field misalignment angles Φ_*x*_ and Φ_*y*_, applied in the *x*-*z* and *y*-*z*-planes, respectively. For the small angles we investigated, ${{\Phi }}\approx {({{{\Phi }}}_{x}^{2}+{{{\Phi }}}_{y}^{2})}^{1/2}$. As expected, when Φ = 0, no nuclear FID contrast is observed, because in this case ***B***_eff_ and ***e***_init_ are both collinear with ***e***_*z*_ such that the nuclear spin is optically pumped into the non-precessing eigenstate $\left\vert 0,+1/2\right\rangle$ of ${\hat{H}}^{{{{\rm{gs}}}}}$. Upon increasing Φ, ***B***_eff_ and ***e***_init_ both tilt away from ***e***_*z*_ in different directions, resulting in nuclear precession of increasing contrast *C*. At the same time, increasing Φ (i.e., *B*_⊥_) tends to reduce the nuclear hyperpolarization efficiency^28,29^ and the NV optical spin readout contrast^16^, both of which reduce *C*. Overall, these counteracting effects imply that there is an optimal tilt angle Φ_opt_ which maximizes *C* by balancing the magnitude of the nuclear spin coherences with nuclear spin readout efficiency. We call this maximized contrast ${C}_{\max }$.

To determine Φ_opt_ and ${C}_{\max }$, we show in Fig. 2b the data from Fig. 2a as a function of transverse field *B*_⊥_, where for each data point, we determined *B*_⊥_ from the NV’s full optically detected magnetic resonance spectrum (see Supplementary Notes C). Figure 2b reveals a clear maximum in *C* at *B*_⊥_ ≈ 8.6 G, which corresponds to Φ_opt_ ≈ 0.86^∘^. These results are in good agreement with the predictions of our numerical model (black curve in Fig. 2b). The quality of the simulation depends sensitively on the NV intersystem crossing rates, all of which were kept fixed to literature values^16^ in our calculations (see supplementary notes A). We assign remaining discrepancies between data and simulations to uncertainties on optical transition rates employed in the model.

Interestingly, we find that our all-optical ^15^N nuclear FID protocol is relatively resilient to deviations of ***B***_ext_ away from ideal ESLAC conditions. For this, we investigated the dependance of the contrast *C* on the applied magnetic field ∣***B***_ext_∣ and tilt angle Φ_*x*_, where for each data point, we ensured that Φ_*y*_ = 0 is maintained to within experimental accuracy. The resulting data show a nontrivial dependance of *C* on ∣***B***_ext_∣ and Φ_*x*_ (Fig. 2c), and in particular reveal that both Φ_opt_ and ${C}_{\max }$ change with ∣***B***_ext_∣ (Fig. 2d). These dependencies are qualitatively captured by our numerical model. We find a global maximum of ${C}_{\max }\approx 4.2\, \%$ for ∣***B***_ext_∣ = 533 G, and a drop of *C* over a full-width at half maximum (FWHM) range of ~50 G.

### Nuclear spin precession frequency

**Fig. 3:**
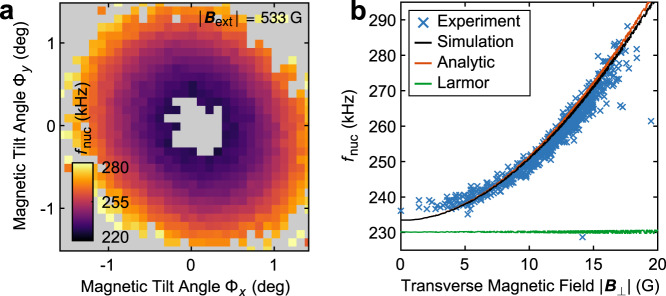
Investigation of nuclear FID frequency. **a** ^15^N nuclear precession frequency *f*_nuc_ corresponding to the data shown in Fig. 2a. Only pixels for which *C* > 1% are shown. **b** Same data as in **a**, plotted against total transverse magnetic field *B*_⊥_, together with the numerical model prediction (black), the prediction from Eq. (5) (orange), and the bare Larmor frequency *γ*_*I*_*B*_*z*_ (green).

Further, we investigate the dependance of *f*_nuc_ on the magnetic field tilt angles Φ_*x*_ and Φ_*y*_. For this, we determine *f*_nuc_ by Fourier analysis of the FID data for each data point sampled in Fig. 2a. The result is shown in Fig. 3a, along with the corresponding plot of the same data as a function of ∣*B*_⊥_∣ in Fig. 3b. The precession frequency *f*_nuc_ increases with *B*_⊥_ in a way that is excellently described by both Eq. (5) and our numerical model. We again assign small discrepancies between measured and predicted values of *f*_nuc_ to experimental uncertainties in determining ***B***_ext_.

### Nuclear coherence time

**Fig. 4:**
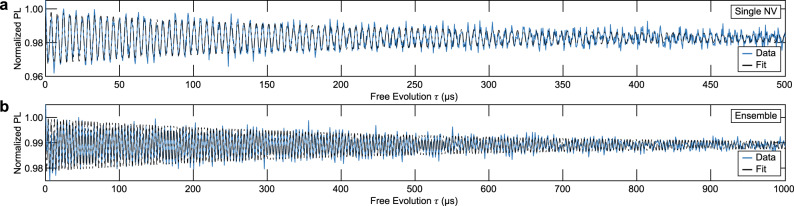
Nuclear FID coherence time. All-Optically measured nuclear spin precession of **a**, a single NV in a diamond nanopillar, and **b**, an ensemble of NVs in bulk diamond, both measured at ∣***B***_ext_∣ = 533G with tilt angles Φ = 0.65^∘^ and Φ = 0. 8^∘^, for **a** and **b** respectively. Each data set is fitted with a damped harmonic function to determine the nuclear spin coherence time ${T}_{2}^{* }$, yielding ${T}_{2}^{* }=248.1\pm 12.4\,\mu\text{s}$ and ${T}_{2}^{* }=508.5\pm 17.4\,\mu\text{s}$, for **a** and **b** respectively.

The ease of use of our all-optical ^15^N FID experiments enables facile assessment of the ^15^N inhomogeneous nuclear spin coherence time ${T}_{2}^{* }$. To determine ${T}_{2}^{* }$, we extend the measurement pulse sequence shown in Fig. 1c to longer FID evolution times *τ*, to resolve the full decay of the signal (Fig. 4a). Fitting an exponentially decaying harmonic function to the data yields *C* = 3.09 ± 0.11% and ${T}_{2}^{* }=248.1\pm 12.4\,\mu\text{s}$ for the single NV center under investigation. This decoherence time is somewhat shorter than previously reported values^38^, but consistent with the rather short NV electron spin relaxation time of *T*_1_ = 315 ± 16 μs for our shallow NV—a timescale which is known to limit the NV’s nuclear spin decoherence time^39,40^.

### Scaling to NV ensembles

An interesting question with particular relevance for potential applications in quantum sensing is whether our all-optical scheme also scales to ensembles of NV centers. To address this question, we repeated our experiments on an ensemble of NV centers using an unstructured, CVD grown diamond sample with a preferential orientation of NV centers along one of the four possible crystal directions^41^ (see methods). We maximize the contrast *C* in the same fashion as done in the single NV case (see supplementary notes D), and then determine ${T}_{2}^{* }$ through the full FID trace shown in Fig. 4b. Using a least-squares fit as before, we find *C* = 2.08 ± 0.04%, and ${T}_{2}^{* }=508.5\pm 17.4\,\mu\text{s}$ for this NV ensemble. While *C* is comparable to the single NV case (with a slight deterioration due to the minority of non-aligned NVs), ${T}_{2}^{* }$ almost doubles. This value of ${T}_{2}^{* }$, however, is still short of the best-reported values of ≈ 2.2 ms for ^15^NV nuclear spin coherence times^26,38^. We exclude electron spin *T*_1_ relaxation as a source for this fast nuclear spin decoherence, as we measured *T*_1_ = 5.8 ± 0.5 ms in this sample. Possible other sources for nuclear spin dephasing in our ensemble experiment include fluctuations in external magnetic field or temperature^38,40^.

## Discussion

Our all-optical ^15^N FID scheme lends itself to applications in quantum sensing, e.g., in magnetometry and gyroscopy (rotational sensing). In the following, we discuss the predicted performance of such all-optical coherent quantum sensing schemes.

The shot noise limited FID sensitivity for spin based, low-frequency magnetometry is given by^17,42,43^

6 $${\eta }_{{{{\rm{mag}}}}}\approx \frac{1}{\gamma C\sqrt{N{T}_{2}^{* }}}\,.$$

Here, *N* is the average number of detected photons per readout pulse, *C* is the readout contrast, and *γ* is the gyromagnetic ratio of the spins employed for sensing. Further sensitivity reductions due to overhead in preparation and measurement of quantum states are not included in this expression, but are of little relevance to our conclusion, given the long ${T}_{2}^{* }$ times at hand.

Evaluating Eq. (6) for our single NV data (${T}_{2}^{* }=250\,\mu\text{s}$, *C* = 4%, *N* = 0.1) using the effective nuclear gyromagnetic ratio *γ* = 1.2*γ*_*I*_ determined from the slope of the data in Fig. 1e (or from Eq. (5)), we obtain a photon shot noise limited magnetometry sensitivity of *η*_mag_ = 154 μT Hz^−1/2^. Further, given that our approach scales to NV ensembles, we make predictions on future ensemble NV magnetometry sensitivity. For this, we assume a laser power of 100 mW, a 350 ns readout window, and a conversion ratio of excitation photons to detected PL photons of 3.4%^44^, to obtain *N* = 3.2 × 10^9^. Together with the measured ensemble values ${T}_{2}^{* }=500\,\mu\text{s}$ and *C* = 2%, we obtain ${\eta }_{{{{\rm{mag}}}}}^{{{{\rm{ensemble}}}}}=1.22$ nT Hz^−1/2^.

For spin-based gyroscopy, the sensitivity is determined in analogy to Eq. (6), but with omission of the gyromagnetic ratio, i.e., *η*_gyro_ = *γ* ⋅ *η*_mag_^28^. Nuclear spins are therefore particularly attractive for gyroscopy, since their long ${T}_{2}^{* }$ times generally offer them better sensitivities compared to electron spins, while they are less susceptible to magnetic fields and their fluctuations. Employing the same procedure as before, we obtain a projected ensemble gyroscope sensitivity of ${\eta }_{{{{\rm{gyro}}}}}^{{{{\rm{ensemble}}}}}\approx 13{5}^{\circ }\,{{{{\rm{hour}}}}}^{-1/2}$.

To place these estimates in context, we note that best-reported magnetometry sensitivities using electron spin ensembles in diamond were ${\tilde{\eta }}_{{{{\rm{mag}}}}}^{{{{\rm{ensemble}}}}} < 10$ pT Hz^−1/2^ ^45^, while NV-based nuclear spin gyroscopes have recently achieved sensitivities ${\tilde{\eta }}_{{{{\rm{gyro}}}}}^{{{{\rm{ensemble}}}}}=28{0}^{\circ }\,{{{{\rm{hour}}}}}^{-1/2}$ ^26^. We also note briefly that NV-based spin gyroscopes in general are still an emerging technology and not yet competitive with established technologies^26,46^.

While for magnetometry, our projected sensitivity alone is not competitive with the state-of-the-art, the microwave-free modality we present still lends itself to specific applications, e.g., remote sensing through optical fibers^47^, or for cases where the MW drive would critically affect the sample of interest. Conversely, for gyroscopy, we project numbers competitive with previous NV-based approaches. The added feature of all-optical operation is hereby a key asset, which may enable future integrated and power-efficient NV gyroscopes.

Looking forward, we note that our all-optical nuclear spin sensing scheme is also amenable to alternate high-fidelity readout schemes to increase measurement contrast, based on spin-to-charge conversion^48,49^. Another potential path to improving contrast *C* is to dynamically pulse the field misalignment angle between spin initialization and readout, to separately optimize the two processes.

In conclusion, we have presented an all-optical scheme for observing FID dynamics of ^15^N nuclear spins in diamond NV centers. Our technique is based on the optical pumping of the ^15^N nuclear spin into a quantum superposition state—a process that occurs near the NV’s ESLAC in the presence of a small transverse magnetic field. These results may find applications in various fields of quantum sensing, most notably all-optical magnetometry and gyroscopy, for which we give benchmark comparisons that compare favorably with the state-of-the-art. Our results also suggest possible extensions to a range of other relevant scenarios, including analogous dynamics near the NV’s ground-state level anti-crossing, or all-optical addressing of nearby ^13^*C* nuclear spins. The nuclear spin dynamics we discussed should generally be observable in any color center exhibiting suitable level anti-crossing dynamics and coupling to nuclear spins, and might as such offer interesting opportunities for sensing with and characterization of novel color centers in a variety of solid-state hosts.

## Methods

### Single NV diamond sample

The majority of our experimental results (Figs. 1–3 and Fig. 4a) were obtained on a single NV center that was created in an “electronic grade” diamond sample (Element Six) by ion implantation and subsequent sample annealing^50^. For implantation, we employed singly charged ^15^N ions at a flux of 10^11^ cm^−2^ and an energy of 6 keV, corresponding to a nominal implantation depth of ~9 nm^51^. To increase PL collection efficiency, parabolic diamond pillars were fabricated on the diamond surface^52^ subsequent to NV creation. A single pillar containing an individual NV center was studied in this work.

### NV ensemble diamond sample

The NV ensemble sample used to obtain the data shown in Fig. 4b was grown on a CVD diamond substrate along the (113) crystal orientation to facilitate Nitrogen incorporation and create NVs preferentially oriented along the NV axis lying closest to the growth plane^41^. A 15 μm thick layer containing NVs was obtained using ^12^C and ^15^N enriched gas mixture, which led to an estimated NV density of ~300 ppb and a P1-center density of ~0.1 ppm^53^.

### Experimental setup

A home-built confocal microscope (Olympus LMPLFLN-100 objective, NA = 0.8) was used to focus a green laser (Cobolt 06-MLD; emission wavelength 515 nm) on the sample and to simultaneously collect the emitted red PL. All data shown in this paper were taken by optically exciting the NV(s) near saturation, which for our setup corresponded to a laser power of ~50 μW for the single NV in a nanopillar, and 2.2 mW for the ensemble of NVs in unstructured diamond. A static magnetic field was applied using a permanent neodymium disk magnet (supermagnete, 2× S-60-05-N) mounted on a linear translation stage, to tune the magnetic field strength at the NV location. For precise magnetic field alignment near the ESLAC, the magnet is mounted on a goniometric stage (SmarAct SGO-60.5 and SGO-77.5). Finally, the laser and photon detectors were gated with pulses which were created and synchronized using a high-frequency signal generator (Zurich Instruments SHFSG), which also served as a source for microwave pulses used for the characterization of the magnetic field via optically detected magnetic resonance experiments.

### Supplementary information

SUPPLEMENTARY INFORMATION All-Optical Nuclear Quantum Sensing using Nitrogen-Vacancy Centers in Diamond

## Data Availability

The simulated and experimentally acquired data, and the source code for the simulations that support the findings of this study are available at Zenodo^54^.
