# Supplementary material for: All-optical nuclear quantum sensing using nitrogen-vacancy centers in diamond
Source: npj Quantum Inf. 2023 Jun 10;9(1):56. doi: 10.1038/s41534-023-00724-6 (PMC11041803; doi:10.1038/s41534-023-00724-6)
Supplement: Supplementary file 1 — SUPPLEMENTARY INFORMATION All-Optical Nuclear Quantum Sensing using Nitrogen-Vacancy Centers in Diamond [file 41534_2023_724_MOESM1_ESM.pdf]

SUPPLEMENTARY INFORMATION
All-Optical Nuclear Quantum Sensing using Nitrogen-Vacancy Centers in Diamond

B. Bürgler,[1,*] T. F. Sjolander,[1,*] O. Brinza,[2] A. Tallaire,[2,3] J. Achard,[2] and P. Maletinsky[1,†]

[1]*Department of Physics, University of Basel, Klingelbergstrasse 82, Basel CH-4056, Switzerland*

[2]*Laboratoire des Sciences des Procédés et des Matériaux, LSPM, CNRS-UPR 3407, Université Sorbonne Paris Nord, 99 Avenue JB Clément, Villetaneuse 93430, France*

[3]*Institut de Recherche de Chimie Paris, CNRS, Chimie ParisTech, Université PSL, 11 rue Pierre et Marie Curie, 75005 Paris, France*

(Dated: May 23, 2023)

CONTENTS

I. Supplementary Methods 3
A. Effective Nuclear Hamiltonian 3
B. Numerical Model for NV Optical Pumping 5
1. Mathematical Description of the Model 5
2. Matrix Representations of Superoperators 8
3. Details on Simulation Evaluation 10

II. Supplementary Notes 11
A. Comparison of Rate Constant Parameter Sets 11
B. Orientation of the Effective Magnetic Field 13
C. Experimental Determination of the Magnetic Field and the NV Zero Field Splitting 14
D. Ensemble Diamond Data 17

References 17

* These authors contributed equally.
† patrick.maletinsky@unibas.ch

I. SUPPLEMENTARY METHODS

A. Effective Nuclear Hamiltonian

Here, we calculate an effective Hamiltonian for the ^{15}N spin in the electronic $m_S = 0$ subspace. To that end, we employ Van Vleck perturbation theory, which is applicable to any Hamiltonian $\hat{H}$ that can be written in the form $\hat{H} = \hat{H}_0 + \hat{V}$, where $\hat{H}_0$ is block diagonal, made of distinct subspaces, and V is a perturbation that couples the initially uncoupled different subspaces of $\hat{H}_0$. Following the notation in [1], to second order, the effective Hamiltonian for each individual subspace is given by

$$\begin{aligned}\langle i|\,\hat{H}^{\alpha}_{\text{eff}}\,|j\rangle = &\langle i,\alpha|\,H_0 + V\,|j,\alpha\rangle \\ &+ \frac{1}{2}\sum_{k,\gamma\neq\alpha} \langle i,\alpha|\,V\,|k,\gamma\rangle\,\langle k,\gamma|\,V\,|j,\alpha\rangle \times \left[\frac{1}{E_{i,\alpha}-E_{k,\gamma}} + \frac{1}{E_{k,\alpha}-E_{k,\gamma}}\right].\end{aligned} \tag{1}$$

Here, latin indices denote states within a given subspace, and greek indicies count over the subspaces. Equation (1) is valid if the energy difference between states in different blocks is much larger than the coupling between them, $|E_{i,\alpha} - E_{j,\beta}| \gg \langle i,\alpha|\,V\,|j,\beta\rangle$.

Hamiltonian $\hat{H}^{\text{gs}}$ from the main text is such a block diagonal Hamiltonian. Written in the basis $\{|m_S, m_I\rangle\}$, it reads

$$\frac{\hat{H}^{\text{gs}}}{h} = \begin{bmatrix} D_0^{\text{gs}} + \gamma_S B_z + \frac{\gamma_I B_z + A_\parallel^{\text{gs}}}{2} & \frac{\gamma_I}{2} B_x & \frac{\gamma_S}{\sqrt{2}} B_x & 0 & 0 & 0 \\ \frac{\gamma_I}{2} B_x & D_0^{\text{gs}} + \gamma_S B_z - \frac{\gamma_I B_z + A_\parallel^{\text{gs}}}{2} & \frac{1}{\sqrt{2}} A_\perp^{\text{gs}} & \frac{\gamma_S}{\sqrt{2}} B_x & 0 & 0 \\ \frac{\gamma_S}{\sqrt{2}} B_x & \frac{1}{\sqrt{2}} A_\perp^{\text{gs}} & +\frac{1}{2}\gamma_I B_z & \frac{\gamma_I}{2} B_x & \frac{\gamma_S}{\sqrt{2}} B_x & 0 \\ 0 & \frac{\gamma_S}{\sqrt{2}} B_x & \frac{\gamma_I}{2} B_x & -\frac{1}{2}\gamma_I B_z & \frac{1}{\sqrt{2}} A_\perp^{\text{gs}} & \frac{\gamma_S}{\sqrt{2}} B_x \\ 0 & 0 & \frac{\gamma_S}{\sqrt{2}} B_x & \frac{1}{\sqrt{2}} A_\perp^{\text{gs}} & D_0^{\text{gs}} - \gamma_S B_z + \frac{\gamma_I B_z - A_\parallel^{\text{gs}}}{2} & \frac{\gamma_I}{2} B_x \\ 0 & 0 & 0 & \frac{\gamma_S}{\sqrt{2}} B_x & \frac{\gamma_I}{2} B_x & D_0^{\text{gs}} - \gamma_S B_z - \frac{\gamma_I B_z - A_\parallel^{\text{gs}}}{2} \end{bmatrix} \tag{2}$$

where the blocks are defined by states of equal values of m_S, and where, without loss of generality, we define the direction of the transverse magnetic field as the x-direction, e.g. that $B_y = 0$.

We now evaluate Supplementary Eq. (1) for $\hat{H}^{\text{gs}} = \hat{H}_0 + \hat{V}$ with $\hat{H}_0 = D_0^{\text{gs}}\hat{S}_z^2 + \gamma_S b_z \hat{S}_z + \gamma_I b_z \hat{I}_z$ and $\hat{V} = B_\perp(\gamma_I \hat{I}_x + \gamma_S \hat{S}_x) + \hat{\boldsymbol{S}} \cdot \boldsymbol{A} \cdot \hat{\boldsymbol{I}}$, and α corresponding to the $m_S = 0$ subspace, and where we denote $B_x =: B_\perp$. This leads to the following matrix elements,

$$\frac{\langle 1|\,\hat{H}_{\rm eff}^{m_S=0}\,|1\rangle}{h} = \frac{+\gamma_I B_z}{2} + \frac{(A_\perp^{\rm gs})^2}{A_\parallel^{\rm gs} - 2D_0^{\rm gs} + 2\gamma_I B_z - 2\gamma_S B_z} + \frac{4\gamma_S^2 B_\perp^2 D_0^{\rm gs}}{(A_\parallel^{\rm gs} - 2D_0^{\rm gs} + 2\gamma_S B_z)(A_\parallel^{\rm gs} + 2D_0^{\rm gs} + 2\gamma_S B_z)}\ , \tag{3}$$

$$\frac{\langle 2|\,\hat{H}_{\rm eff}^{m_S=0}\,|2\rangle}{h} = \frac{-\gamma_I B_z}{2} + \frac{(A_\perp^{\rm gs})^2}{A_\parallel^{\rm gs} - 2D_0^{\rm gs} - 2\gamma_I B_z + 2\gamma_S B_z} + \frac{4\gamma_S^2 B_\perp^2 D_0^{\rm gs}}{(A_\parallel^{\rm gs} - 2D_0^{\rm gs} - 2\gamma_S B_z)(A_\parallel^{\rm gs} + 2D_0^{\rm gs} - 2\gamma_S B_z)}\ , \tag{4}$$

$$\frac{\langle 1|\,\hat{H}_{\rm eff}^{m_S=0}\,|2\rangle}{h} = \frac{+\gamma_I B_\perp}{2} + \frac{\gamma_S B_\perp A_\perp^{\rm gs}/2}{A_\parallel^{\rm gs} - 2D_0^{\rm gs} + 2\gamma_S B_z} + \frac{\gamma_S B_\perp A_\perp^{\rm gs}/2}{A_\parallel^{\rm gs} - 2D_0^{\rm gs} - 2\gamma_S B_z} + \frac{\gamma_S B_\perp A_\perp^{\rm gs}/2}{A_\parallel^{\rm gs} - 2D_0^{\rm gs} + 2\gamma_S B_z - 2\gamma_I B_z} + \frac{\gamma_S B_\perp A_\perp^{\rm gs}/2}{A_\parallel^{\rm gs} - 2D_0^{\rm gs} - 2\gamma_S B_z + 2\gamma_I B_z} \tag{5}$$

$$\frac{\langle 2|\,\hat{H}_{\rm eff}^{m_S=0}\,|1\rangle}{h} = \frac{\langle 1|\,\hat{H}_{\rm eff}^{m_S=0}\,|2\rangle}{h}\ . \tag{6}$$

Since $\gamma_I \ll \gamma_S$, we simplify the term $(\gamma_S \pm \gamma_I) \approx \gamma_S$ in all denominators. In similar fashion, we use the fact that $A_\parallel^{\rm gs} \ll D_0^{\rm gs}$ and thus set $(A_\parallel^{\rm gs} \pm 2D_0^{\rm gs}) \approx \pm 2D_0^{\rm gs}$ in all denominators. We obtain the following effective Hamiltonian for the ^{15}N spin in the $m_S = 0$ manifold:

$$\frac{\hat{H}_{\rm eff}^{m_S=0}}{h} = \frac{1}{2}\begin{bmatrix} +\gamma_I B_z + \frac{+\gamma_S B_z (A_\perp^{\rm gs})^2 - D_0^{\rm gs}(A_\perp^{\rm gs})^2 - 2D_0^{\rm gs}(\gamma_S B_\perp)^2}{(D_0^{\rm gs})^2 - (\gamma_S B_z)^2} & \gamma_I B_\perp - \frac{2\gamma_S B_\perp A_\perp^{\rm gs} D_0^{\rm gs}}{(D_0^{\rm gs})^2 - (\gamma_S B_z)^2} \\ \gamma_I B_\perp - \frac{2\gamma_S B_\perp A_\perp^{\rm gs} D_0^{\rm gs}}{(D_0^{\rm gs})^2 - (\gamma_S B_z)^2} & -\gamma_I B_z + \frac{-\gamma_S B_z (A_\perp^{\rm gs})^2 - D_0^{\rm gs}(A_\perp^{\rm gs})^2 - 2D_0^{\rm gs}(\gamma_S B_\perp)^2}{(D_0^{\rm gs})^2 - (\gamma_S B_z)^2} \end{bmatrix}. \tag{7}$$

Next, we add an energy-offset of $(D_0^{\rm gs}(A_\perp^{\rm gs})^2 - 2D_0^{\rm gs}(\gamma_S B_\perp)^2)\,/\,((D_0^{\rm gs})^2 - (\gamma_S B_z)^2)$ to place the energy levels symmetrically around zero, and thereby obtain the Hamiltonian given in the main text,

$$\frac{\hat{H}_{\rm eff}^{m_S=0}}{h} = \frac{1}{2}\begin{bmatrix} \gamma_I B_z + \nu_z & \gamma_I B_\perp + \nu_\perp \\ \gamma_I B_\perp + \nu_\perp & -\gamma_I B_z - \nu_z \end{bmatrix}. \tag{8}$$

where

$$\nu_z = \frac{\gamma_S B_z (A_\perp^{\rm gs})^2}{(D_0^{\rm gs})^2 - (\gamma_S B_z)^2} \tag{9}$$

denotes the correction to the diagonal elements caused by mixing between states of different m_S, and

$$\nu_\perp = \frac{-2\,\gamma_S B_\perp A_\perp^{\rm gs} D_0^{\rm gs}}{(D_0^{\rm gs})^2 - (\gamma_S B_z)^2} \tag{10}$$

is the corresponding correction to the off-diagonal elements. Note that these expressions for ν_z and $\nu_\perp$ are diverging for $D_0^{\mathrm{gs}} = \gamma_S B_z$, e.g. near the ground state level anti-crossing. However, Van Vleck formalism is not applicable to that regime since the corresponding electronic subspaces of H_0 are not sufficiently spaced in energy once this condition is approached.

B. Numerical Model for NV Optical Pumping

In this section, we present in detail our numerical model for simulating the spin dynamics of the NV center with and without green illumination for a given magnetic field $\boldsymbol{B}$. To calculate the trajectory of both the electron and nuclear spin under optical pumping it is necessary to consider not only classical rate equations coupling the orbital states, but also to incorporate the quantum mechanical evolution of the spins within each orbital state. Our model follows an approach previously taken to model the effect of chemical reaction kinetics on NMR spectra [2].

1. Mathematical Description of the Model

We model the room temperature NV^- center as a system made up of three distinct electronic states: The ground state (gs), the excited state (es), and a meta stable singlet state (s). We neglect the distinct E_x and E_y orbital branches in the excited state as they are efficiently averaged at room temperature, as well as the existence of two singlet states – we assume that there is only one such singlet state. We also neglect laser induced ionization to the NV^0 state.

First we define a separate spin density operator for each orbital state (labelled with α) which evolves coherently per the Liouville-von Neumann equation of motion

$$\frac{\mathrm{d}}{\mathrm{d}t}\hat{\rho}_\alpha = \hat{\hat{L}}_\alpha \hat{\rho}_\alpha, \tag{11}$$

where the carets denote operators, double carets denote superoperators, and α indexes over the different orbital states. The commutation superoperator $\hat{\hat{L}}_\alpha$ in Equation (11) can be calculated from the corresponding Hamiltonian $\hat{H}_\alpha$ as

$$\hat{\hat{L}}_\alpha = -i\left(\hat{H}_\alpha \otimes E_\alpha - E_\alpha \otimes \hat{H}_\alpha^T\right), \tag{12}$$

D_0^{gs}	[MHz]	+2870.760402
$A_{\parallel}^{\text{gs}}$	[MHz]	+3.03
$A_{\perp}^{\text{gs}}$	[MHz]	+3.65
D_0^{es}	[MHz]	+1420
$A_{\parallel}^{\text{es}}$	[MHz]	−57.8
$A_{\perp}^{\text{es}}$	[MHz]	−39.2
γ_S	[MHz/G]	0.000431744
γ_I	[MHz/G]	2.802494716

Supplementary Table I. Numeric values for the employed NV parameters, taken from [3, 4].

where $\alpha \in \{\text{es}, \text{gs}, \text{s}\}$ denotes the orbital, E_α is the identity matrix of the same dimensionality as H_α, and T denotes matrix transposition. The Hamiltonians for each orbital state are given by:

$$\hat{H}^{\text{gs,es}}/h = D_0^{\text{gs,es}} \hat{S}_z^2 + \hat{\boldsymbol{S}} \cdot \boldsymbol{A}^{\text{gs,es}} \cdot \hat{\boldsymbol{I}} + \gamma_S \boldsymbol{B} \cdot \hat{\boldsymbol{S}} + \gamma_I \boldsymbol{B} \cdot \hat{\boldsymbol{I}} \tag{13}$$

$$\hat{H}^{\text{s}}/h = \boldsymbol{B} \cdot \hat{\boldsymbol{\sigma}}\,. \tag{14}$$

where $\hat{\boldsymbol{S}}$ and $\hat{\boldsymbol{I}}$ are angular momentum operators for the electron and ^{15}N nucleus acting on the joint electron/nuclear Hilbert space, and $\hat{\boldsymbol{\sigma}}$ are the 2x2 spin-1/2 matrices. The constants and coupling tensors are defined in Supplementary Table I. Next, we couple these differential equations with additional (real valued) superoperators corresponding to the incoherent optical pumping process. These superoperators act to reduce or increase the population of a given spin state and thus take the role of spin-selective relaxation superoperators

$$\begin{aligned}
\frac{\mathrm{d}}{\mathrm{d}t}\hat{\rho}_{\text{gs}} &= \hat{\hat{L}}_{\text{gs}}\hat{\rho}_{\text{gs}} - k_{\text{green}}\hat{\rho}_{\text{gs}} + k_{\text{red}}\hat{\rho}_{\text{es}} + k_{\text{s1}}(\hat{\hat{S}}_{+1}^{\otimes} + \hat{\hat{S}}_{-1}^{\otimes})\hat{\rho}_s + k_{\text{s0}}\hat{\hat{S}}_0^{\otimes}\hat{\rho}_{\text{s}} \\
\frac{\mathrm{d}}{\mathrm{d}t}\hat{\rho}_{\text{s}} &= \hat{\hat{L}}_{\text{s}}\hat{\rho}_{\text{s}} - (2k_{\text{s1}} + k_{\text{s0}})\hat{\rho}_{\text{s}} + k_{\text{ISC}}^{m_s=|1|}\hat{\hat{T}}^e(\hat{\hat{P}}_1 + \hat{\hat{P}}_{-1})\hat{\rho}_{\text{es}} + k_{\text{ISC}}^{m_s=0}\hat{\hat{T}}^e\hat{\hat{P}}_0\hat{\rho}_{\text{es}} \\
\frac{\mathrm{d}}{\mathrm{d}t}\hat{\rho}_{\text{es}} &= \hat{\hat{L}}_{\text{es}}\hat{\rho}_{\text{es}} + k_{\text{green}}\hat{\rho}_{\text{gs}} - k_{\text{red}}\hat{\rho}_{\text{es}} - k_{\text{ISC}}^{m_s=|1|}(\hat{\hat{P}}_{+1} + \hat{\hat{P}}_{-1})\hat{\rho}_{\text{es}} - k_{\text{ISC}}^{m_s=0}\hat{\hat{P}}_0\hat{\rho}_{\text{es}},
\end{aligned} \tag{15}$$

where $\hat{\hat{P}}_{\pm 1,0}$ are projection superoperators that project $\hat{\rho}_\alpha$ onto the NV-electron spin state with $m_S = \pm 1, 0$ while leaving the dimensionality of $\hat{\rho}_\alpha$ unchanged. This superoperator ensures that the rate of inter system crossing (ISC) out of the excited state depends on the

instantaneous spin state population of $\hat{\rho}_{\mathrm{es}}$. Next, $\hat{\hat{T}}^e$ is a partial trace superoperator that acts on a 36-dimensional joint electron/nuclear density operator and traces out the NV-electron degrees of freedom, leaving a 4-dimensional density operator corresponding only to ^{15}N. Finally, $\hat{\hat{S}}^{\otimes}_{0,\pm1}$ is a direct product superoperator that acts on a 4-dimensional ^{15}N density operator and turns it into a joint electron/nuclear density operator with the NV-electron in the state $ms = 0, \pm1$. Note that because their effect changes dimensionality of $\hat{\rho}_\alpha$, the matrix representations of $\hat{\hat{T}}^e$ and $\hat{\hat{S}}^{\otimes}_{0,\pm1}$ are not square. Further, since none of these superoperators act on the ^{15}N degrees of freedom, this model assumes that the nuclear spin state is preserved throughout the optical pumping process. Equation (15) is normalized such that the sum of the traces of the spin density operators $\hat{\rho}_\alpha$ is equal to 1, meaning Trace$\{\hat{\rho}_\alpha\}$ is the fractional population of the total system in orbital state α. We will give procedures for generating the corresponding matrix representations for the superoperators in Supplementary Eq. (15) later in this section.

Figure 1 shows a pictorial representation of the processes modeled by Supplementary Eq. (15). The ground and excited states are coupled via optical excitation with rate k_{green} and radiative decay with rate k_{red} respectively. The spin selective ISC causes non-radiative transitions from the excited state to the singlet, described by the rates $k_{\mathrm{ISC}}^{m_s=\pm1,0}$. Relaxation from the electronic singlet into the ground state is also electron spin selective, with rates given by k_{s1} and k_{s0} respectively. The precise values for the ISC and relaxation rates have been subject to considerable debate. At the end of these section we present modeling results for different sets of these parameters.

By concatenating the vector representations of $\hat{\rho}_{\mathrm{gs}}$, $\hat{\rho}_{\mathrm{s}}$, and $\hat{\rho}_{\mathrm{es}}$ into a single 76x1 spin density operator for the entire system, $\hat{\rho}$, Supplementary Eq. (15) can conveniently be cast in matrix form:

$$\frac{\mathrm{d}}{\mathrm{d}t}\begin{bmatrix}\hat{\rho}_{\mathrm{gs}}\\ \hat{\rho}_{\mathrm{s}}\\ \hat{\rho}_{\mathrm{es}}\end{bmatrix} = \begin{bmatrix}\hat{\hat{L}}_{\mathrm{gs}} - k_{\mathrm{green}}\hat{\hat{E}}_{36} & +k_{s\pm1}(\hat{\hat{S}}^{\otimes}_{1} + \hat{\hat{S}}^{\otimes}_{-1}) + k_{s0}\hat{\hat{S}}^{\otimes}_{0} & +k_{\mathrm{red}}\hat{\hat{E}}_{36}\\ 0 & \hat{\hat{L}}_{s} - (2k_{s\pm1} + k_{s0})\hat{\hat{E}}_4 & +k_{ISC}^{m_S=|1|}\hat{\hat{T}}^e(\hat{\hat{P}}_1 + \hat{\hat{P}}_{-1}) + k_{\mathrm{ISC}}^{m_S=0}\hat{\hat{P}}_0\\ k_{\mathrm{green}}\hat{\hat{E}}_{36} & 0 & \hat{\hat{L}}_{es} - k_{\mathrm{red}}\hat{\hat{E}}_{36} - k_{\mathrm{ISC}}^{m_S=|1|}(\hat{\hat{P}}_1 + \hat{\hat{P}}_{-1}) - k_{\mathrm{ISC}}^{m_S=0}\hat{\hat{P}}_0\end{bmatrix}\begin{bmatrix}\hat{\rho}_{gs}\\ \hat{\rho}_{s}\\ \hat{\rho}_{es}\end{bmatrix}, \quad (16)$$

where $E_{36(4)}$ is a 36x36(4x4) identity matrix. In the following, we will call the resulting 76x76 dimensional matrix $\hat{\hat{A}}$. Note that because of how we formed $\hat{\rho}$ by concatenation it cannot represent coherences between different orbital states, however such coherences are unlikely to be significant and are not necessary to explain the physics of interest in this work. We also note that since we use identity superoperators to represent the radiative processes,

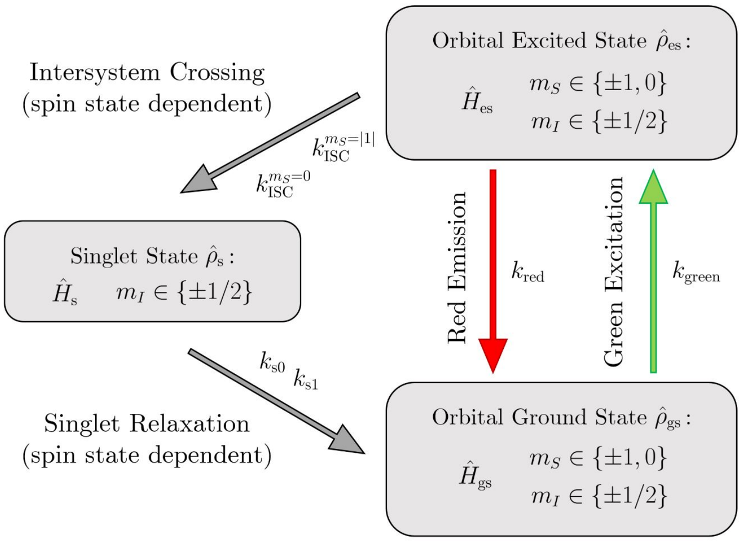

Supplementary Figure 1. **Level Structure for the optical pumping model.** Each of the three orbital states is governed by its own Hamiltonian, and these states are coupled by four different processes: the intersystem crossing, singlet relaxation, excitation with green light and relaxation under emission of red PL. Excited and ground state have both electron and nuclear spin degrees of freedom, while the singlet state exhibits only nuclear spin degrees of freedom.

spin-spin coherences will be preserved under optical excitation and radiative decay within this model. Since the matrix $\hat{\hat{A}}$ commutes with itself for all values of time t, Supplementary Eq. (16) can easily be integrated and thus the time evolution of the system can be calculated as

$$\hat{\rho}(t) = \mathrm{e}^{2\pi\hat{\hat{A}}t}\hat{\rho}(0), \tag{17}$$

for any time t. At any given time the first 36 entries of $\hat{\rho}(t)$ corresponds to $\hat{\rho}_{gs}(t)$ written in vector form, the next 4 entries are $\hat{\rho}_s(t)$ and the last 36 are $\hat{\rho}_{es}(t)$. We take the predicted instantaneous photoluminescence for $\hat{\rho}(t)$ as the fractional population in the excited electronic state,

$$\mathrm{PL}(t) = \mathrm{Trace}\{\hat{\rho}_{es}(t)\}\,. \tag{18}$$

2. *Matrix Representations of Superoperators*

We define the matrix representations of the ^{15}N spin operators on the joint electron/nuclear space as $\hat{I}_{x/y/z} = \hat{\sigma}_{x/y/z} \otimes \hat{E}_3$ and the NV-electron spin operators as $\hat{S}_{x/y/z} = \hat{E}_2 \otimes \hat{\lambda}_{x/y/z}$,

where $\hat{\lambda}$ are the 3x3 spin-1 matrices. The action of the partial trace superoperator can be understood by considering $\hat{\hat{T}}^e\left(\hat{\rho}_I \otimes \hat{\rho}_S\right) = \text{Trace}\left\{\hat{\rho}_S\right\} \cdot \hat{\rho}_I$, where $\hat{\rho}_{I(S)}$ describes the 2(3) dimensional density operator of the nuclear(electron) subspace. The matrix representation of $\hat{\hat{T}}^e$ is determined following the procedure laid out in [2] and reproduced here for completeness:

$$\{\hat{\hat{T}}^e\}_{\alpha\beta} = \begin{cases} 1 & \text{, if } m = n \\ 0 & \text{, else} \end{cases} \tag{19}$$

where

$$\begin{aligned} \alpha &= (i-1) \cdot d_I + j \\ \beta &= (((i-1) \cdot d_S + m) - 1) \cdot d_S \cdot d_I + (j-1) \cdot d_S + n\,, \end{aligned} \tag{20}$$

where $i, n = 1 \ldots d_I$ count through the degrees of freedom of the first subspace (nuclear in our case), and $j, m = 1 \ldots d_S$ count through the degrees of freedom of the second subspace (NV-electron in our case). The resulting $\hat{\hat{T}}^e$ matrix is 4x36 dimensional.

The action of the direct product superoperator $\hat{\hat{S}}^{\otimes}_{0,\pm1}$ can be understood by considering $\hat{\hat{S}}^{\otimes}_{0,\pm1}\hat{\rho}_I = \hat{\rho}_I \otimes \hat{\rho}_{m_S=0,\pm1}$, where $\hat{\rho}_{m_S=0,\pm1}$ describes the density operator corresponding to a electron $m_S = \{0, \pm1\}$ eigenstate. The matrix representation of the direct product superoperator is

$$\{\hat{\hat{S}}^{\otimes}_{0,\pm1}\}_{\alpha\beta} = \{\hat{\kappa}_{0,\pm1}\}_{mn} \tag{21}$$

where

$$\begin{aligned} \alpha &= (((i-1) \cdot d_S + m) - 1) \cdot d_S \cdot d_I + (j-1) \cdot d_S + n \\ \beta &= (i-1) \cdot d_I + j\,, \end{aligned} \tag{22}$$

and

$$\hat{\kappa}_{+1} = \begin{pmatrix} 1 & 0 & 0 \\ 0 & 0 & 0 \\ 0 & 0 & 0 \end{pmatrix} \qquad \hat{\kappa}_{0} = \begin{pmatrix} 0 & 0 & 0 \\ 0 & 1 & 0 \\ 0 & 0 & 0 \end{pmatrix} \qquad \hat{\kappa}_{-1} = \begin{pmatrix} 0 & 0 & 0 \\ 0 & 0 & 0 \\ 0 & 0 & 1 \end{pmatrix}. \tag{23}$$

The numbers i, j, n, m, d_S and d_I are the same as defined above. The resulting dimensionality of the matrix representation of $\hat{\hat{S}}^{\otimes}_{0,\pm1}$ is 36x4.

Finally, we define the matrix representation for the projection superoperator,

$$\hat{\hat{P}}_{0,\pm1} = \sum_{k=1}^{4} \rho^k_{0,\pm1} \cdot (\rho^k_{0,\pm1})^T \tag{24}$$

where $\rho^k_{0,\pm1} = \hat{\kappa}^k_{\text{nuc}} \otimes \hat{\kappa}_{0,\pm1}$ is the 36x1 column vector representation of the joint 6x6 density matrix operator and

$$\hat{\kappa}^1_{\text{nuc}} = \begin{pmatrix} 1 & 0 \\ 0 & 0 \end{pmatrix} \qquad \hat{\kappa}^2_{\text{nuc}} = \begin{pmatrix} 0 & 1 \\ 0 & 0 \end{pmatrix} \qquad \hat{\kappa}^3_{\text{nuc}} = \begin{pmatrix} 0 & 0 \\ 1 & 0 \end{pmatrix} \qquad \hat{\kappa}^4_{\text{nuc}} = \begin{pmatrix} 0 & 0 \\ 0 & 1 \end{pmatrix} . \tag{25}$$

While $\hat{\hat{T}}^e$ and $\hat{\hat{S}}^{\otimes}_{0,\pm1}$ both change the dimensionality of the state they operate on, $\hat{\hat{P}}_{0,\pm1}$ is a square 36x36 matrix and thus preserves dimensionality. The sum in equation (24) ensures that $\hat{\hat{P}}_{0,\pm1}$ projects onto a particular electronic spin state, while leaving the nuclear spin unchanged.

3. *Details on Simulation Evaluation*

In order to evaluate Supplementary Eq. (17), we use literature values for the various NV spin-Hamiltonian NV parameters and the optical transition rates and inter-system-crossing rates, with the exception of D^{gs}_0 which we determine experimentally as described in the Supplementary notes on the magnetic field map. The exact values for the spin Hamiltonian parameters are listed in Supplementary Table I and are taken from [3, 4]. There appears to be less consensus regarding the precise values of the optical transition rates and the inter-system-crossing rates. Therefore, we consider five different sets of rate constants, labeled parameter sets 1 to 5, as shown in the Supplementary Table II.

In order to simulate the spin dynamics in the experiments, we calculate the time evolution of the spin system using Supplementary Eq. (17) both in the presence and the absence of green illumination by setting the parameter $k_{\text{green}} = s \cdot k_{\text{red}}$ or $k_{\text{green}} = 0$ respectively. Here, $s = 0.35$ is the saturation parameter which we determined experimentally on the single NV sample, and k_{red} is given by the employed optical model. The full propagation is done piecewise using different $\hat{\hat{A}}$ matrices. As the starting point of the simulation we take $\hat{\rho}_{gs}(0) = (1/6)\,\hat{E}_6$, and $\hat{\rho}_{es}(0) = 0$, and $\hat{\rho}_s(0) = 0$, corresponding to an initial state where all the population is in the electronic ground state, which is entirely non-polarized. The optical initialization step was 3 µs and the readout step was 0.350 µs. For the readout step we calculated the evolution in timesteps of 1 ns (thus simulating the PL transient curve) and took the final PL value as the sum of the 350 individual values. The time τ that the system is allowed to evolve with $k_{\text{green}} = 0$ between the initialization and the readout is swept in order to simulate PL(τ), the observable in our experiments.

	Parameter Set 1	Parameter Set 2	Parameter Set 3	Parameter Set 4	Parameter Set 5
k_{red} [MHz]	66	77	62.70	63.2	67.4
$k_{\text{ISC}}^{m_S=0}$ [MHz]	0	0	12.97	10.8	9.9
$k_{\text{ISC}}^{m_S=\|1\|}$ [MHz]	57	30	80.00	60.7	96.6
k_{s0} [MHz]	1.0	3.3	3.45	0.8	4.83
k_{s1} [MHz]	0.7	0	1.08	0.4	1.055
Reference	[5]	[6]	[7]	[8]	[9]

Supplementary Table II. Numeric values for of the optical transition rates used in our model. We consider five different parameter sets. In the main text, we use set 4.

We analyze the results by taking the Fourier transform of $\text{PL}(\tau)$. The oscillation frequency f is the maximimum at non-zero frequency, and the simulated contrast is calculated as $C = 4V/V_0$, where V is the amplitude of the peak at f, and V_0 is the amplitude of the peak at zero frequency.

II. SUPPLEMENTARY NOTES

A. Comparison of Rate Constant Parameter Sets

In order to investigate the impact on the simulation results of different choices for the rate constants we simulated ^{15}N nuclear FIDs for the full range of experimental conditions discussed in the main text for each of the parameter sets in Supplementary Table II. The numerically predicted contrast C as a function of off-axis magnetic field at $|\boldsymbol{B}_{\text{ext}}| = 533\,\text{G}$ is shown in Supplementary Figure 4a, where for ease of comparison, the experimental data has been normalized to 1 and the simulation to 0.75. Depending on parameter set we find maximal contrast for $B_\perp$ between 7.9 and 10.2 G. In Supplementary Figure 4b we show the maximal contrast C_{max} as a function of $|\boldsymbol{B}_{\text{ext}}|$. All rate constant parameter sets predict a global maximum in C between 530 and 550 G. We note that sets 1 and 2, for which $k_{\text{ISC}}^{m_S=0} = 0$, redict a second local maximum for C at about 470 G, however there is no such indication of a second maximum in the experimental data.

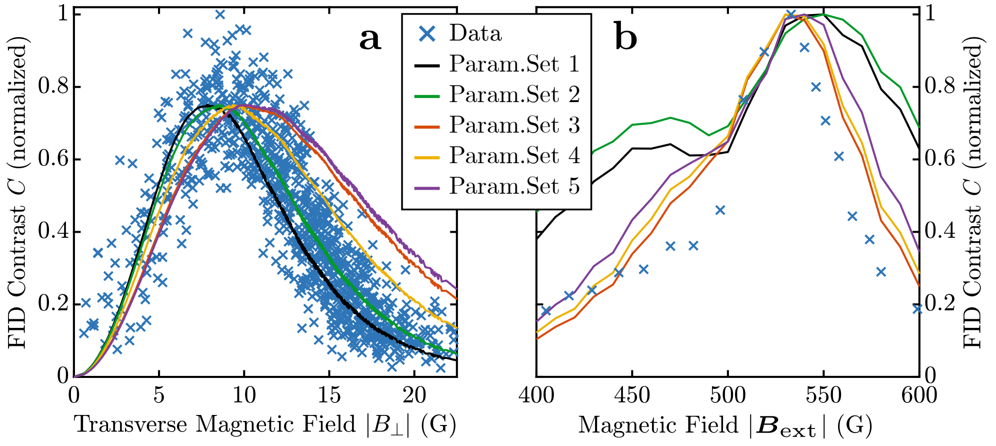

Supplementary Figure 2. **Numerical simulation results for different optical transition rate models.** Here, we present the experimental data shown in Fig 2b and d in the main text (blue crosses), together with the results of the numerical simulation, run with each of the five optical transition rate parameter sets (solid lines). We deem set 4 to have the best agreement with the data, which is why model 4 is used for all numerical results shown in the main text.

Based on these two simulations we find that parameter set 4 has the best agreement with our data; the corresponding curve only shows a single maximum in Supplementary Figure 4b, while at the same time in Supplementary Figure 4a, the line corresponding to set 4 stays closer to the experimental data cloud than either of sets 3 or 5. We thus use set 4 for all the simulations presented in the main text.

The main result of of our work is that under certain magnetic field conditions, starting from a completely unpolarized mixed state, optical pumping alone is enough to initialize the nuclear spin of the NV center into a coherent superposition state with respect to the external magnetic field. Our numerical model predicts that the state ρ_{init} of the ^{15}N spin after initialization is fairly insensitive to the precise values of the rate constants that govern the NV optical pumping dynamics. Quantitative results are shown in Supplementary Figure 3. As in the main text, $\boldsymbol{e}_{\mathrm{init}}$ is defined as the expectation value of the nuclear spin operator $\hat{\boldsymbol{I}}$ of the $\hat{H}_{\mathrm{es}}$ eigenvector with the greatest $m_S{=}0$ character. Further, $\hat{\rho}_{\mathrm{init}}$ is calculated as the projection onto the nuclear spin operator of $\hat{\rho}_{\mathrm{gs}}$ after the 3 µs optical initialization pulse plus an additional 50 ns evolution step with $k_{\mathrm{green}} = 0$ to ensure that residual ρ_{es} population decays to the ground state. It is clear from Supplementary Figure 3 that no matter the rate constant parameter set chosen ρ_{init} is roughly aligned with $\boldsymbol{e}_{\mathrm{init}}$, and if the ISC rate for the

$m_s = 0$ states is 0 then ρ_{init} will be nearly collinear with $\boldsymbol{e}_{\text{init}}$. Meanwhile, if $k_{\text{ISC}}^{m_S=0} \neq 0$ the predicted initialization fidelity is slightly worse.

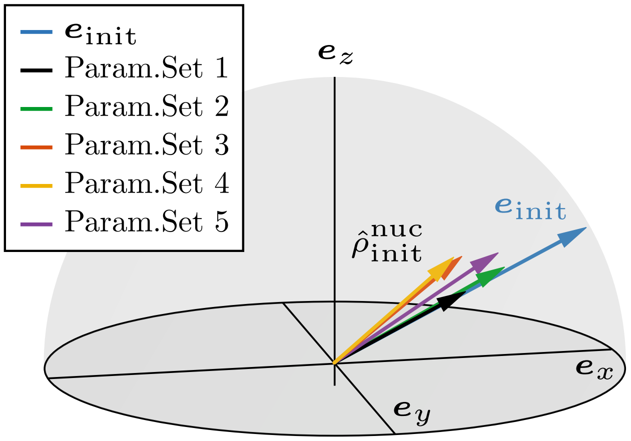

Supplementary Figure 3. **Numerical simulations showing the dependance of ρ_{init} on different optical transition rate models.**

B. Orientation of the Effective Magnetic Field

In this work we show that near the ESLAC the ^{15}N nuclear spin precesses around around an effective magnetic field that is strongly tilted away from the externally applied magnetic field due to the influence of the hyperfine interaction with the NV electron spin. In this section we show that the angle ϑ by which $\boldsymbol{B}_{\text{eff}}$ is tilted away from the NV axis can be calculated in three ways, using either the analytical model based on Van Vleck perturbation theory or by direct diagonalization of the full ground state spin Hamiltonian, or by propagating the system using the numerical model described in Supplementary Methods. In the main text we showed that using Van Vleck perturbation theory we have $\vartheta = \tan^{-1}\left[(\gamma_I B_\perp + \nu_\perp)/(\gamma_I B_z + \nu_z)\right]$.
Alternatively, diagonalization of the full H_{gs} for a given magnetic field orientation yields 6 eigenvectors. Of these 6 eigenvectors we take the one with the largest $|m_S = 0, m_I = 1/2\rangle$ character and in this case we calculate a normalized vector, $\boldsymbol{V}_{\text{num}}$, from the expectation value of the nuclear spin operator $\boldsymbol{I}$ of this eigenvector. With this method we have $\vartheta = \cos^{-1}\left[\boldsymbol{e}_z \cdot \boldsymbol{V}_{\text{num}}\right]$
Finally, it is also possible to infer ϑ from the trajectory of $\rho(\tau)$ predicted by the numerical model presented in Supplementary Methods. In this case we evaluated the trajectory of the

expectation value of the nuclear spin operator as $\left\langle \hat{\boldsymbol{I}}(\tau) \right\rangle = \text{Trace}\{\hat{\boldsymbol{I}} \cdot \rho(\tau)\}$ for each time point during the free evolution period of a simulated nuclear FID experiment and fit a plane to the resulting data. We then calculated a normalized vector, $\boldsymbol{V}_{\text{sim}}$, by taking the cross product between two randomly chosen vectors in this plane. With this method we have $\vartheta = \cos^{-1}[\boldsymbol{e}_z \cdot \boldsymbol{V}_{\text{sim}}]$

In Supplementary Figure 4 we show a plot of the angle of $\boldsymbol{B}_{\text{eff}}$ with respect to the NV axis, plotted against the angle of the applied magnetic field with respect to the NV axis. It is clear that the approximations made in the perturbation theory treatment are valid in this regime, the three results are in good agreement.

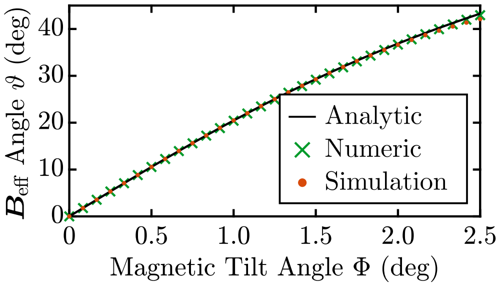

Supplementary Figure 4. **Comparison of tilt angle ϑ predictions.** The predicted angle ϑ with respect to the NV axis of the effective magnetic field around which the 15N spin precesses in the all optical nuclear FID experiments. The angle is calculated using both the analytical aproach based on perturbation theory presented in the main text, numerical diagonalization of the full ground state spin Hamiltonian, and inferred from the simulated trajectory of the density matrix, calculated using the numerical model presented in Supplementary Methods.

C. Experimental Determination of the Magnetic Field and the NV Zero Field Splitting

In the following we elaborate on how we obtained the values for $B_\perp$ and D_0^{gs} used in the plots throughout this work. To control the external magnetic field we used a permanent magnet mounted on a 2-axis goniometer. However since the distance between the magnet-goniometer assembly and the diamond is used to set the strength of the $\boldsymbol{B}_{\text{ext}}$ bias field, the center of rotation of the goniometer will not in general be precisely at the position of the NV center for a given value of $\boldsymbol{B}_{\text{ext}}$. Therefore, it is in general not entirely accurate to

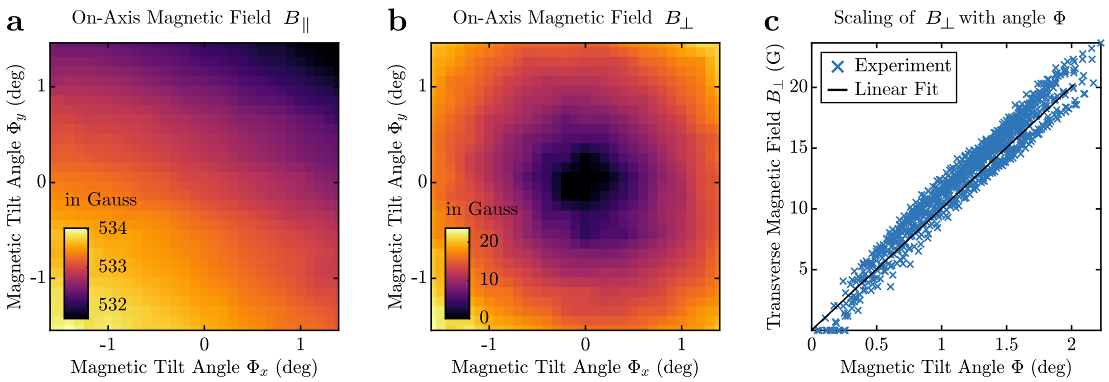

Supplementary Figure 5. **Magnetic field map based on ODMR measurements. a** Parallel magnetic field component $B_{\|}$ and **b** transverse magnetic field component $B_{\perp}$ as a function of tilt angles Φ_x and Φ_y, measured via optically detected magnetic resonance. Each of the shown pixels corresponds to one pixel in the main text's Fig. 2a and 3a. The total magnetic field is about $|\boldsymbol{B}_{\text{ext}}| = 533$ G. **c** Measured transverse magnetic field as a function of total tilt angle Φ, revealing a linear dependance with slope 10.01 G/deg, allowing for a simple conversion of $B_{\perp}$ to corresponding Φ and vice versa.

simply measure $|\boldsymbol{B}_{\text{ext}}|$ and set $B_{\perp} = \sin(\Phi) \cdot |\boldsymbol{B}_{\text{ext}}|$, since the rotation itself will also (slightly) change the distance between the magnet and the diamond. Most of the data in this paper were obtained at a bias field of $\boldsymbol{B}_{\text{ext}} = 533$ G. For this field only we carefully measured the full $\boldsymbol{B}$ vector as a function of the two goniometer angles.

The first step of the procedure was to measure the contrast C of the ^{15}N FID signal as described elsewhere in this work as a function of the two goniometer angles. The resulting data are shown in Figure 2a in the main text. We know that for the goniometer orientation corresponding to the center pixel where C goes to zero, the magnetic field is perfectly aligned with the NV axis, i.e. $B_{\perp} = 0$ for this pixel. Knowing this we measured the full magnetic resonance spectrum of the NV center on this pixel, and fit the resulting 2 transitions (2 transitions only because under these conditions the ^{15}N is fully polarized) to the ground state nuclear spin Hamiltonian $\hat{H}_{\text{gs}}$ with $B_{\|}$ and D_0^{gs} as free parameters, enforcing the condition that $B_{\perp} = 0$. From this fit we obtained the value for the zero field splitting for this particular NV center as $D_0^{\text{gs}} = 2870.760402$ MHz. In the second step we measured ODMR spectra as a function of the two goniometer angles, and fit the measured 4 (or 2 depending on hyperpolarization levels) transitions to $\hat{H}_{\text{gs}}$ with $B_{\|}$ and $B_{\perp}$ as the

free parameters. Finally, we interpolate both $B_{\parallel}(\Phi_x, \Phi_y)$ and $B_{\perp}(\Phi_x, \Phi_y)$ to twice the pixel density in order to match the experimental data in Fig. 2a and 3a.

The results are shown in Supplementary Figure 5. We also plot $B_{\perp}$ against total tilt angle ($\Phi = (\Phi_x^2 + \Phi_y^2)^{1/2}$), revealing a near linear relationship with slope 10.01 G/deg.

Throughout this work where we plot experimental and simulated data against $B_{\perp}$ (main text figures 2a, 2b, 3a, 3b) we have used the magnetic field map in Supplementary Figure 5 to obtain the conversion from goniometer position to magnetic field. Further in order to make a statement of the ideal magnetic tilt angle corresponding to $B_{\perp} = 8.6$ G in Figure 2b of the main text, we used the slope from Supplementary Figure 5c.

The experimentally measured magnetic field map is valid only at 533 G. Therefore, for data sets where we swept the distance between the magnet and the diamond in order to vary the strength of the bias field (Figures 1e, 2c, 2d) we instead just report the tilt angle as defined directly by the goniometer.

D. Ensemble Diamond Data

In the main text of this work we carefully map the dependance on the nuclear FID contrast on the orientation of the applied magnetic field for single NV center in a nanostructured pillar. In this section we show that the same behavior is reproduced on ensemble samples. Data are shown in Supplementary Figure 6, where we plot the contrast and frequency of the nuclear spin FID as a function of goniometer orientation and bias field strength. The measurements show clearly that the physics described in this work extend to ensembles of NVs. We note, however, that the ensemble shows a contrast C of about half the single-NV contrast, which we assign to the minority of non-aligned NVs.

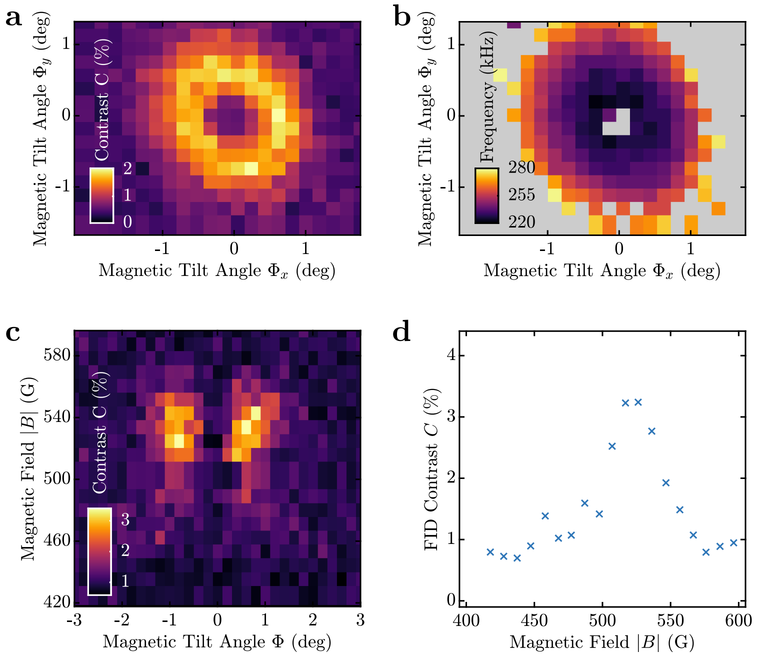

Supplementary Figure 6. **All-Optically detected nuclear precession in an NV ensemble. a** Contrast plotted as a function of magnetic field orientation. The results are qualitatively similar to the single NV case, however we observe slightly lower contrast C. **b** Precession frequency plotted against magnetic field orientation. **c** Contrast plotted against magnetic tilt angle and bias field strength. **d** Maximum contrast for each bias field plotted against bias field strength.

---

[1] Cohen-Tannoudji, C., Dupont-Roc, J. & Grynberg, G. Complement b1. In *Atom-Photon Interactions: Basic Processes and Applications*, page 45 (Wiley, 1998).

[2] Knecht, S., Pravdivtsev, A. N., Hövener, J.-B., Yurkovskaya, A. V. & Ivanov, K. L. Quantitative description of the SABRE process: rigorous consideration of spin dynamics and chemical exchange. *RSC Adv.* **6**, 24470–24477 (2016).

[3] Gali, A. Identification of individual ^{13}C isotopes of nitrogen-vacancy center in diamond by combining the polarization studies of nuclear spins and first-principles calculations. *Physical Review B* **80**, 241204 (2009).

[4] Felton, S. *et al.* Hyperfine interaction in the ground state of the negatively charged nitrogen vacancy center in diamond. *Phys. Rev. B* **79**, 075203 (2009).

[5] Zhang, C. *et al.* Diamond magnetometry and gradiometry towards subpicotesla DC field measurement. *Phys. Rev. Appl.* **15**, 064075 (2021).

[6] Manson, N. B., Harrison, J. P. & Sellars, M. J. Nitrogen-vacancy center in diamond: Model of the electronic structure and associated dynamics. *Phys. Rev. B* **74**, 104303 (2006).

[7] Robledo, L., Bernien, H., Sar, T. v. d. & Hanson, R. Spin dynamics in the optical cycle of single nitrogen-vacancy centres in diamond. *New J. Phys.* **13**, 025013 (2011).

[8] Tetienne, J.-P. *et al.* Magnetic-field-dependent photodynamics of single NV defects in diamond: an application to qualitative all-optical magnetic imaging. *New J. Phys.* **14**, 103033 (2012).

[9] Gupta, A., Hacquebard, L. & Childress, L. Efficient signal processing for time-resolved fluorescence detection of nitrogen-vacancy spins in diamond. *J. Opt. Soc. Am. B* **33**, B28–B34 (2016).
